# On the influence of surface coating on tissue biomechanics – effects on rat bones under routine conditions with implications for image-based deformation detection

**DOI:** 10.1186/s12891-018-2308-z

**Published:** 2018-10-27

**Authors:** Aqeeda Singh, Mario Scholze, Niels Hammer

**Affiliations:** 1 Department of Anatomy, University of Otago, Lindo Ferguson Building, 270 Great King St, Dunedin, 9016 New Zealand; 2 Institute of Materials Science and Engineering, Chemnitz University of Technology, Chemnitz, Germany; 3 Department of Orthopedic and Trauma Surgery, University Clinics of Leipzig, Leipzig, Germany; 4 Fraunhofer IWU, Dresden, Germany

**Keywords:** 3-point bending test, Biomechanical experiment, Chemical fixation, Digital image correlation, Tensile test

## Abstract

**Background:**

Biomechanical testing using image-based deformation detection techniques such as digital image correlation (DIC) offer optical contactless methods for strain and displacement measurements of biological tissues. However, given the need of most samples to be speckled for image correlation using sprays, chemical alterations with impact on tissue mechanicals may result. The aim of this study was to assess the impact of such surface coating on the mechanical properties of rat bones, under routine laboratory conditions including multiple freeze-thaw cycles.

**Methods:**

Two groups of rat bones, highly-uniform and mixed-effects, were assigned to six subgroups consisting of three types of surface coating (uncoated, commercially-available water- and solvent-based sprays) and two types of bone conditions (periosteum attached and removed). The mixed-effects group had undergone an additional freeze-thaw cycle at − 20 degrees. All bones underwent a three-point bending test ranging until material failure.

**Results:**

Coating resulted in similar and non-significantly different mechanical properties of rat bones, indicated by elastic moduli, maximum force and bending stress. Scanning electron microscopy showed more pronounced mechanical alterations related to the additional freeze-thaw cycle, with fewer cracks being present in a bone from the highly-uniform group.

**Conclusions:**

This study has concluded that surface coating with water- or solvent-based sprays for enhancing image correlation for DIC and having an additional freeze-thaw cycle do not significantly alter mechanical properties of rat bones. Therefore, this method may be recommended as an effective way of obtaining a speckled pattern.

## Background

Biomechanical testing is a frequently used tool to assess load deformation behaviour of tissue samples in the field of medicine. The information gathered is utilised for clinical purposes, for example, understanding the mechanical performance of bones allows researchers to predict the effects of pathological processes. It also allows researchers to identify areas of stress that may result in fractures during intense activity [1]. The exact measurement of displacements and strain during deformation is a key point for the determination of accurate material properties. To facilitate this, digital image correlation (DIC) is being used to measure displacement and strain of different materials [2]. This is a contactless optical method of imaging the deformations occurring at the surface of the tissue when loaded, which avoids measurement inaccuracies arising from material slippage with tension tests [3]. Furthermore, DIC measures displacements without the influence of the mechanical properties (and elasticity) of the testing device itself.

The method of DIC typically involves software-based algorithms, which facilitate tracking discrete points on the sample and analysing their relative displacement that has been captured in a series of images during the deformation [3]. DIC is dependent on surfaces which have distinct surface characteristics that can serve as natural speckles. Surfaces which do not have natural contrasts, e.g. tendons and ligaments, need to be speckled and this is commonly done using commercially-available sprays. Using sprays that deposit paint dots randomly is quick and effective method to achieve such speckled appearance [4]. As a consequence of this surface pre-treatment, alterations to the mechanical properties of the biological tissues may result.

It is hypothesised that these changes in properties may be the result of chemical, or osmotic changes in the tissues. The solvent-based spray may increase the elastic modulus and strength of the bones due to chemically and osmotically changing the periosteum. The effect may be explained by the ingredients in the solvent-based spray; acetone and xylene are two key ingredients used in the spray that are also used for tissue fixation. As such, the periosteum of the bone may become a target of the denaturation and dehydration induced by the fixatives as it is a cellular connective tissue. Acetone is commonly used as a dehydrating agent for preparing histological sections, as it physically exchanges with water [5]. Xylene is a nonpolar solvent that is used as a ‘clearing agent’ in preparation of tissue sections, and leads to tissue shrinkage and increases stiffness [6, 7]. The combination of these two ingredients may affect the periosteum (soft tissue) on the bones and consequently result in changes in the mechanical properties.

A second issue may be introduced by using DIC in samples which have been frozen in a non-standardized manner for storage purposes. Freezing avoids the use of fixatives such as formaldehyde or ethanol to prevent autolysis and bacterial contamination [8]. It furthermore helps in acquiring an adequate sample size over a longer period, resulting in tissues with similar properties [9]. However, in ‘real-life’ scenarios, samples that have been gathered may have already undergone additional freeze-thaw cycles beyond the required protocol. It may be that the bones that have undergone additional freeze-thaw cycles show a greater difference between the coated and uncoated conditions due to the formation of ice needles; ice needles can cause structural damage by growing rapidly at − 15 and − 30 °C. This can lead to the deterioration of the mechanical properties in the slow-freezing femora especially if they are frozen at − 20 °C after being thawed, as was the case in a previous study [9, 10]. Commonly also, tests are performed with more than a one-day interval, and therefore require re-freezing between tests as well as the initial freezing for storage [11]. The micro-fractures caused by these ice needles can form pathways for the sprays and consequently result in more marked chemical alterations.

The purpose of this study was to assess the effects of surface coating on the mechanical properties of rat bones (hard tissues), in a standardized scenario and in a second ‘real-life’ scenario with an additional freeze-thaw cycle. The latter approach involving refreezing of tissues is not ideal for any mechanical testing but may form a scenario which researchers can be faced with. It was hypothesised that (A) solvent-based spray on the bones may lead to altered mechanical properties, indicated by an increased strength and elastic modulus due to tissue changes. The secondary hypothesis (B) was that the coated and uncoated bone samples may show a greater difference in the group which had undergone an additional freeze-thaw cycle due to the effects of freezing.

## Methods

### Sample preparation

Bilateral femora and humeri were removed from seventeen rats harvested from animals used for another study approved by the ethics committee of University of Otago (ethical approval number: ET23/16). The animals were euthanized prior to this study for teaching purposes as part of an animal surgery laboratory using CO_2_; following the culling of the animals, the tissues were subsequently precooled at 4 °C for 12 h and then shock frozen at − 80 °C. Thawing was done in the inverted manner: here, the samples were transferred from − 80 °C to − 18 °C before they were transferred to room temperature. The aim of this approach was to minimize the formation of ice needles, in line with studies on ligament-bone complexes showing unaltered mechanics as a consequence of freeze-thaw cycles [12–14]. Before the experiments, the bones were dissected further to remove the extra soft tissue, leaving the periosteum fully intact, and this was carried out in a rapid manner to circumvent any drying. Following the dissection, the bones were placed in isotonic saline (0.9 mass %).

All seventeen rats were of the same species, *Rattus norvegicus*; ten rats were of the breed Wistar (500–600 g, all male), and seven rats were of the breed Sprague Dawley (~ 250 g, male and female). The two breeds were separated into two different groups: highly-uniform (Wistar) and mixed-effects (Sprague Dawley). The mixed-effects Sprague Dawley rat bones group had undergone an additional freeze-thaw cycle to simulate realistic laboratory conditions, whereas the highly-uniform Wistar rat bone group had not, therefore these labels intend to reflect the difference in pre-experimental storage conditions that may affect the end outcome. The highly-uniform Wistar rat bones group also consisted of only male rats, thereby making it more ‘uniform’. The additional freeze-thaw cycle consisted of the mixed-effects group of bones undergoing another cycle of thawing (to room temperature of 21 °C) and freezing (directly at − 20 °C) without any pre-cooling.

### Mechanical testing

**Fig. 1:**
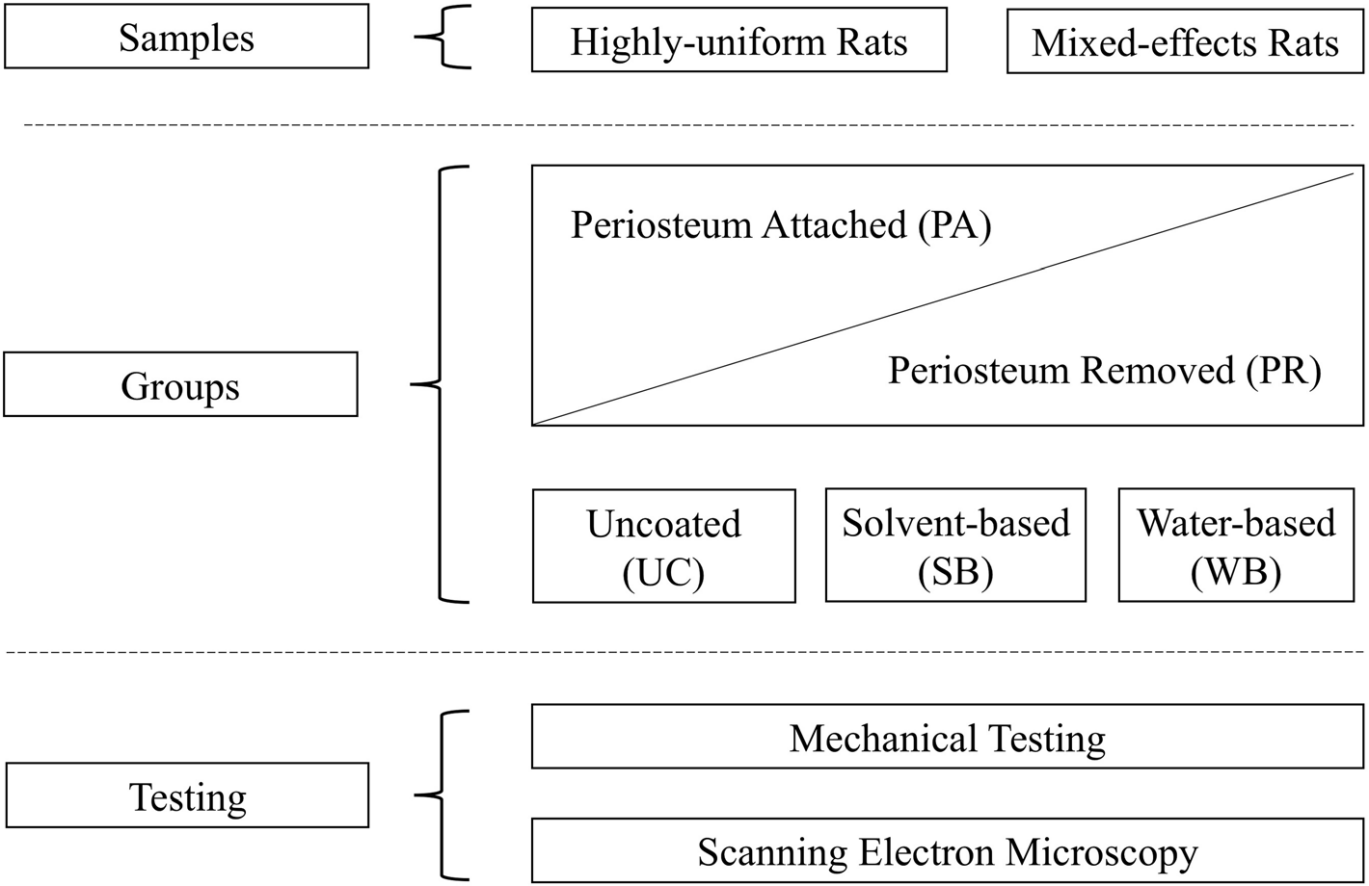
An overview of the experiment

All samples, from both groups of rat femora and humeri, were assigned to subgroups randomly, as seen in Fig. 1, which shows the division of samples for the experiments. Prior to testing, all bone samples were divided into a group with periosteum attached (PA) and another group with periosteum removed (PR). These samples were further split into three subsamples each, depending on the nature of the surface coating of the tissue. One subsample was left uncoated (UC), a second one was coated using a water-based spray (WB), and a third one with a solvent-based spray (SB).

**Fig. 2:**
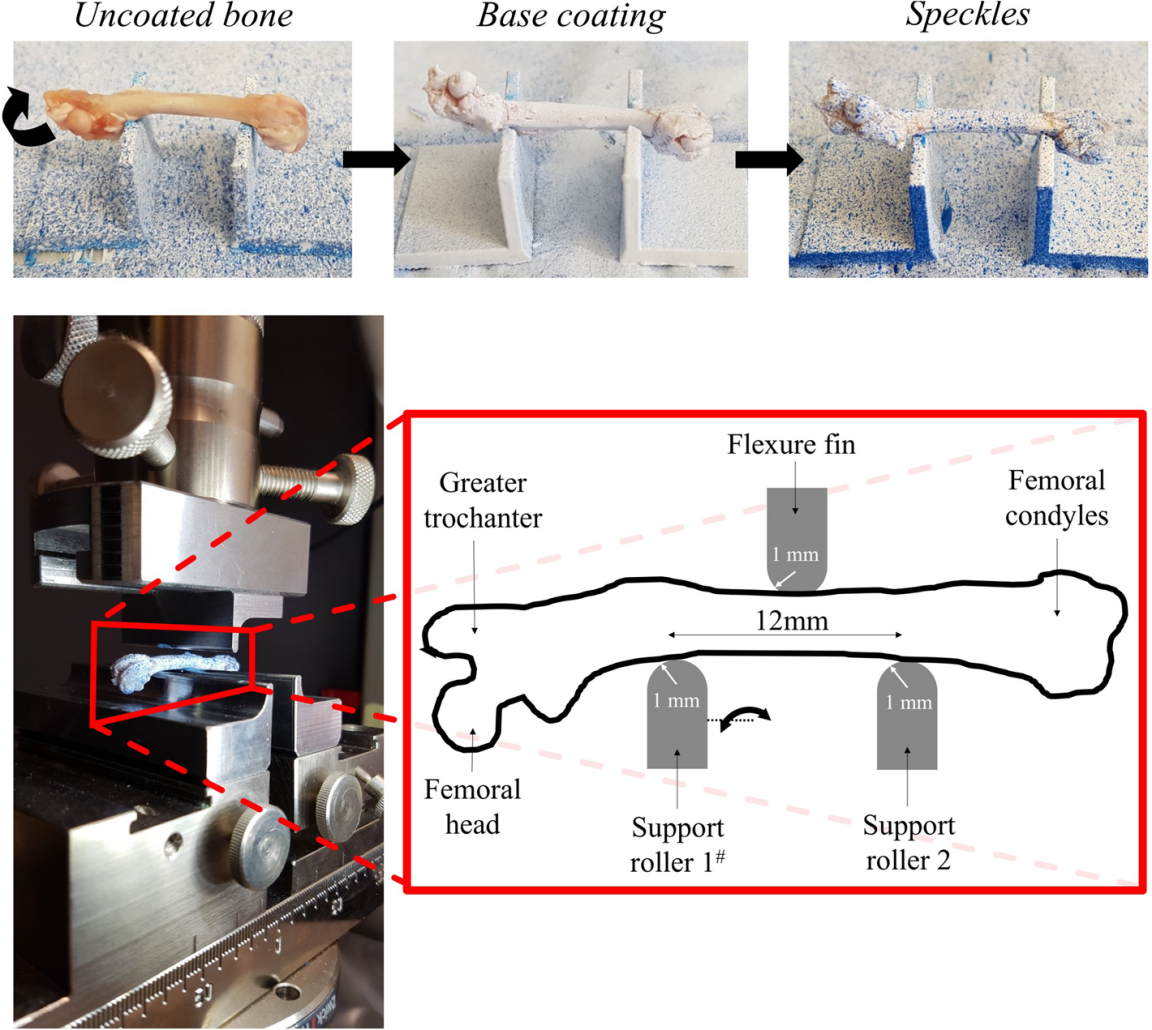
Top: coating sequence represented on a femur (note: the alignment of the femur was changed during the coating process for a homogenous distribution of speckles. The same spray stand was used for all three stages hence the inconsistent background). Bottom: setup three-point bending test, with two flexure fins and a plunger. Crosshead displacement was 10 mm/min. (^#^note that the left side of the support roller had an additional rotational degree of freedom, allowing to adjust for the bones’ outer shape during the tests, as seen in the image)

Five or more femora and five or more humeri were allocated to each of the six subgroups: UC-PA, UC-PR, WB-PA, WB-PR, SB-PA and SB-PR. Each bone was removed from saline, and six diameter measurements (Electronic Digital Vernier Caliper, MechPro, Melbourne, Vic, Australia, +/− 0.02 mm) were made along the axis perpendicular to the shaft of the bone in the mediolateral plane. Always, the maximum diameter was assessed for each area of circumference, and the measurements were taken in 0.5-mm distances before being averaged to calculate a mean diameter and cross-sectional area. To obtain a pattern of dots on the uncoated samples (Fig. 2), a charcoal pencil (Charcoal Pencil Pitt Monochrome, Faber-Castell, Stein, Germany) was used. The water- and solvent-based sprays that were used to create a speckle of dots were provided by Dy-Mark Pty Ltd. (Wacol, QLD, Australia). The ingredients in the sprays are listed in Table 1.

**Table 1:** Ingredients of the sprays used, per the manufacturers’ data sheets

Water-based (WB) spray ingredients	Solvent-based (SB) spray ingredients
Isopropanol (10–30%) Water (10–30%) Dimethyl ether (30–60%) Ammonia (< 1%)	Xylene (10–30%) Acetone (10–30%) Pigment and filler (10–30%) Resin (10–30%) Dimethyl ether (1–10%) Hydrocarbon propellant (10–30%) (less than 0.1% 1, 3 butadiene)

The coated samples were sprayed with the respective white water- or solvent-based spray to create a base coating, then sprayed with the respective blue water- or solvent-based spray to create the distribution of dots to enhance the contrast for image-based deformation detection (Fig. 2). The underlying base coating was spread until a complete coverage of the shaft was reached, followed by the application of small speckles typically with a speckle size of 0.2–0.8 mm in diameter. The spraying was standardised between the samples by placing every sample 20 cm from the spray nozzle and only spraying for a total of ten to fifteen seconds until the bones were sufficiently coated. Each coated sample had a drying period of fifteen minutes after being sprayed before being tested mechanically.

Tests were conducted using a Z020 universal testing device with a Xforce P 2.5 kN load cell and the TestXpert II software (Zwick Roell Group, Ulm, Germany). The machine displacement system was used for the evaluations of all experiments as the displacement of the sample was assumed to equal the crosshead displacement of the test machine, to prevent influences of the different applied spray patterns itself. Image-based deformation detection was carried out using a method described previously in a paper on biomechanical analysis of stiffness and fracture displacement by Höch et al. (2017), to obtain exemplary image-based evaluations [15].

The bones were subjected to three-point bend testing to failure, where the top-oriented surface of the bone-specimen (touching the plunger) was situated in a state of compression and the bottom-oriented surface was in tension (Fig. 2). For the tests, a preload of 2 N was applied prior to the beginning of data recording. Details on the three-point bending test are given in Fig. 2. Force and vertical displacement were captured over time with a sampling rate of 100 Hz.

The maxima of tensile stress and strain in the sample exist at the bottom-oriented surface directly below the point of load application in the bending plane and thus were named bending stress and bending strain. The nominal values were calculated with the assumption of a perfect circular cross section according to

$$ {\sigma}_f=\frac{8 FL}{\pi {d}^3} $$

and

$$ {\epsilon}_f=\frac{6 Dd}{L^2} $$

respectively [16, 17].

*σ*_*f*_=stress in the lowermost (highest tensioned) fibres of the bone directly below plunger (MPa), *F* = load at a given point on the load deflection curve (N), *L* = support span [8], *d* = diameter of the bone [8], *ϵ*_*f*_=strain in the outer surface in the lowermost (highest tensioned) fibres of the bone directly below plunger (mm/mm), *D* = deflection of the centre of the beam [8]. The elastic modulus was evaluated as the slope of the linear part of the bending-stress-bending-strain curve by regression.

**Fig. 3:**
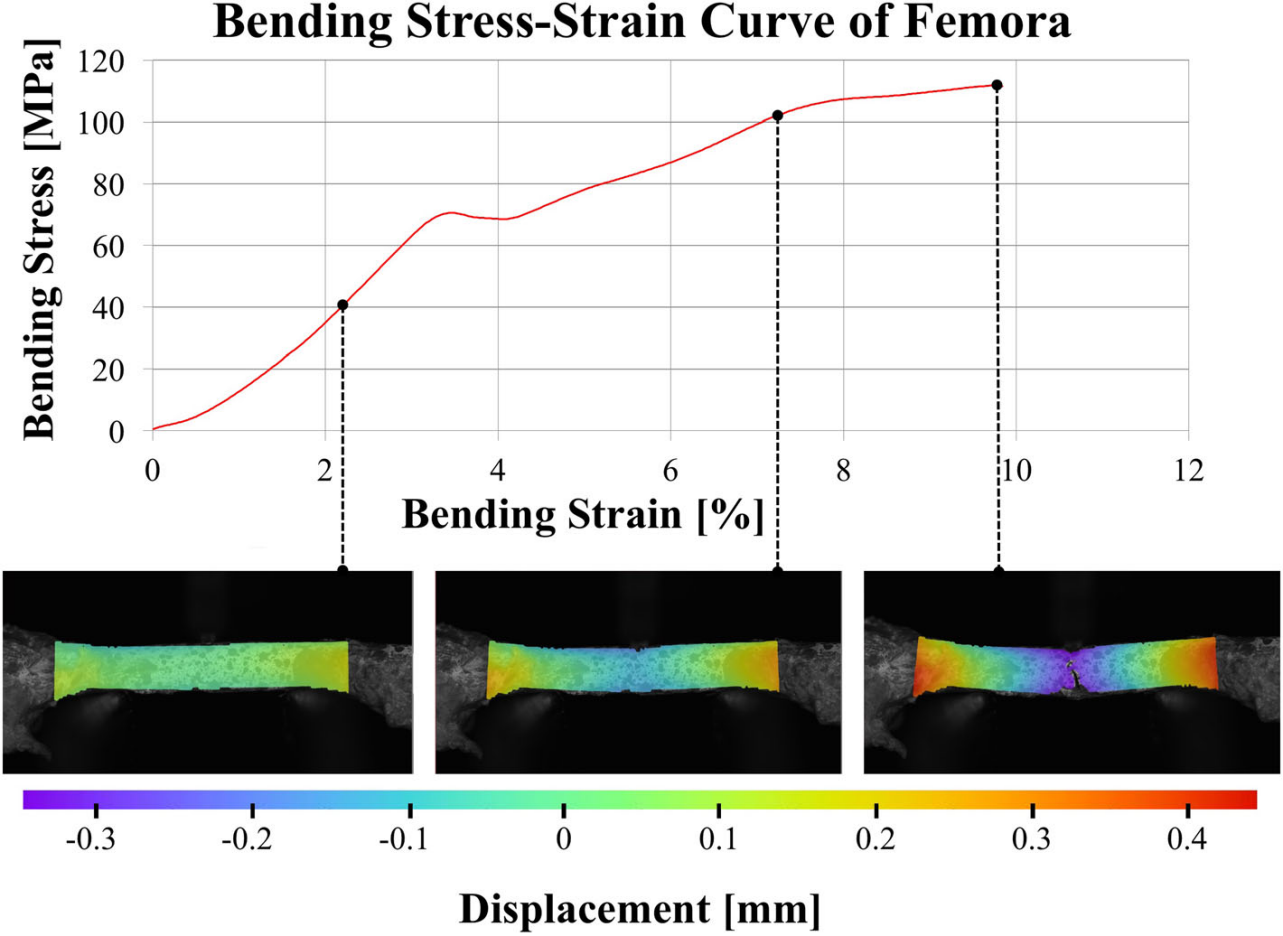
Bending-stress-bending-strain curve in comparison to displacements optically measured using a one-camera setup at different deformations during 3-point bending tests, reaching until material failure, of a water-based (WB) femora with periosteum removed (PR). Note that the fracture line forms distally of the site of the load application (right image)

An example of the combined bending stress-strain and image-based deformation detection data is shown in Fig. 3.

### Scanning Electron microscopy

Six representative femora were chosen to be prepared for the scanning electron microscopy (SEM). Three samples were randomly chosen from the highly-uniform group and three samples from the mixed-effects group. Subsequent to the three-point bending tests, the bones were fixed in 3% neutral-buffered formaldehyde and then post-fixed in 3% glutaraldehyde/0.1 M phosphate buffer (pH = 7.4). Following this, the bones were dehydrated after being washed in distilled water to allow for the critical point drying process (CPD-030, Bal-Tec AG, Liechtenstein) to take place. The magnifications at which SEM was conducted ranged between 25x and 500x using a JSM-6700F field emission microscope (JEOL Ltd., Tokyo, Japan).

### Data evaluation and statistical analysis

A power calculation was not performed to determine an appropriate sample size for the study because, although ideal, it may have been unnecessary given the animals were highly consistent in terms of breed, age and weight. Microsoft Excel (version 15.3, Microsoft Corp., Albuquerque, NM, USA) and Prism 7a (GraphPad Software Inc., Lajolla, CA, USA) were used. The stress-strain data obtained during the femora and humeri experiments were further processed to compute elastic modulus (GPa), maximum force (N), bending strength (MPa), deflection at maximum force [8], strain at maximum force (%), force at break (N) and deflection at break [8] for the bones. Comparisons were made between the six subgroups (UA-PA, UC-PR, WB-PA, WB-PR, SB-PA, SB-PR) in the two different femora and humeri groups. Elastic modulus, maximum force, strength and strain at maximum force were the values being compared statistically. Following assessment for normal distribution, a one-way ANOVA test with post-hoc corrections was used to compare the mean elastic modulus, maximum force, strength and strain at maximum force of each of the six groups within each breed group. The level of significance was set at *p ≤ 0.05*.

## Results

Stress-strain data were obtained from the 34 rat femora and 32 rat humeri. No tissues were excluded due to macroscopically-evident pathology or for any other reasons.

### Coating with water- or solvent-based sprays yielded no significant influence on the mechanical properties of rat bone

The data obtained from both the highly-uniform and mixed-effects bone groups suggested that there was no statistically significant difference between coated and uncoated samples. The only significant difference was between the mean bending strength of UC-PA and WB-PR femora in the highly-uniform group (*p = 0.0354*).

**Fig. 4:**
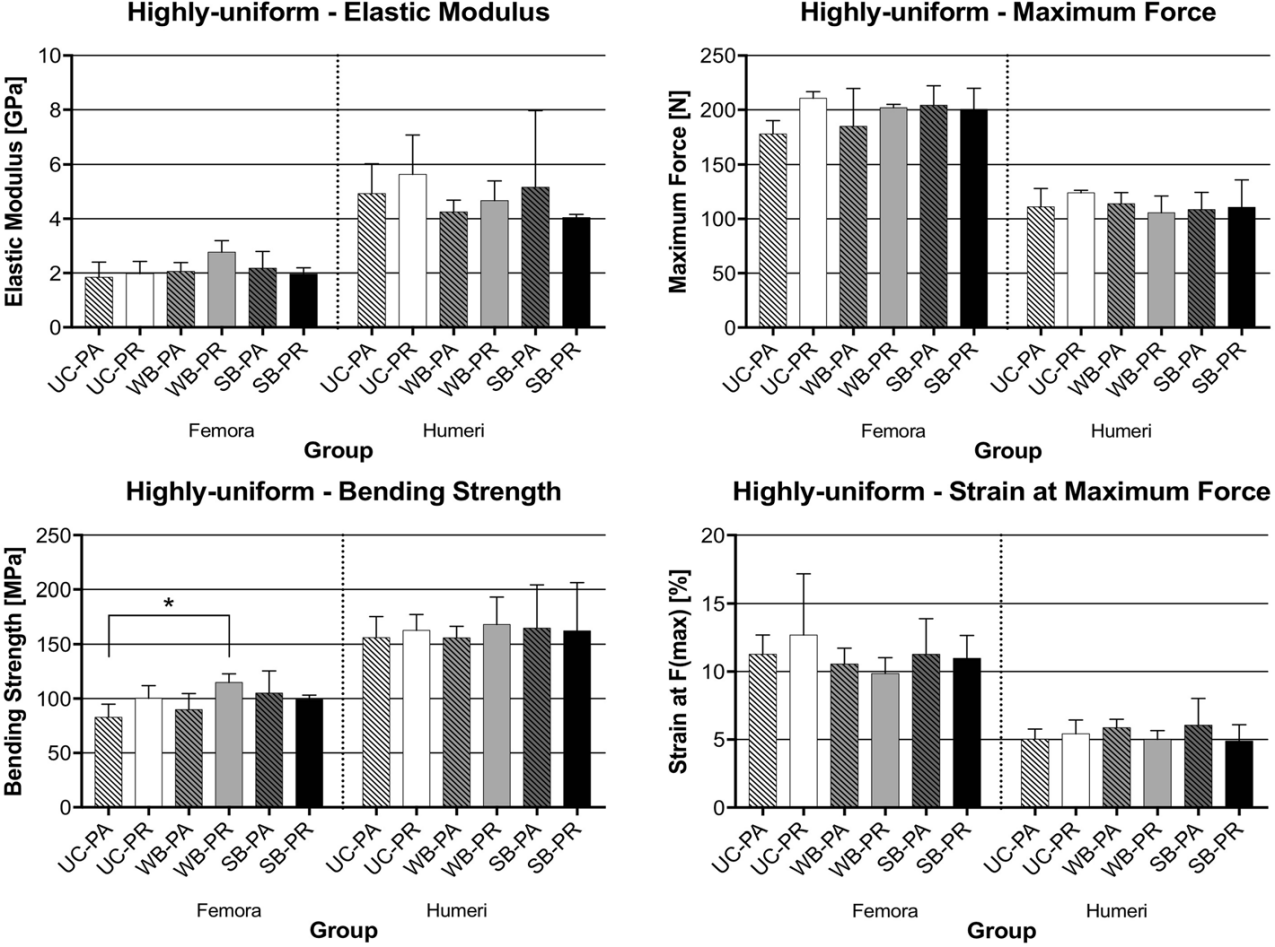
Graphs showing mean values with standard deviations for the highly-uniform group of rat femora and humeri undergoing three-point bending tests (one standardized snap freezing cycle). The significantly different result is indicated with * (*p* = 0.0354)

Further qualitative comparison of the bone groups through the graphs showed minute but statistically non-significant changes. In the highly-uniform rats, the elastic modulus and bending strength tended to be slightly higher in the coated than in the uncoated femora. The mean elastic modulus tended to be slightly higher in samples with the periosteum removed in both femora and humeri for the uncoated and the water-based coating group; these values were non-significantly different and the standard deviation outweighed the differences in mean values. An inverted behaviour was found for the solvent-based group. The maximum force also tended to be slightly higher in femora with periosteum removed for only the uncoated and water-based, with the opposite being true for the solvent-based femora group. Also, the values for elastic modulus, maximum force, bending strength and strain at maximum force were mostly higher in the SB-PA subgroup, compared to the UC-PA subgroup, especially in the highly-uniform bone group. These data are summarised in Table 2 and Fig. 4.

**Table 2:** Summary of the means for the highly-uniform group of rat femora and humeri undergoing three-point bending tests (one standardized snap freezing cycle)

Highly-uniform Group	Elastic Modulus [GPa]	Maximum Force [N]	Bending Strength/Maximum Stress [MPa]	Strain at Maximum Force [%]
	Mean ± Standard Deviation			
Femora				
UC-PA (*n* = 4)	1.85 ± 0.56	178.25 ± 11.87	82.80 ± 12.04	11.27 ± 1.42
UC-PR (*n* = 3)	1.98 ± 0.45	210.69 ± 5.98	100.33 ± 11.58	12.69 ± 4.48
WB-PA (*n* = 3)	2.06 ± 0.32	185.35 ± 34.44	89.85 ± 14.88	10.55 ± 1.17
WB-PR (*n* = 3)	2.77 ± 0.43	202.02 ± 3.00	114.86 ± 7.98	9.86 ± 1.18
SB-PA (n = 4)	2.18 ± 0.61	204.21 ± 17.97	105.03 ± 20.43	11.27 ± 2.61
SB-PR (n = 3)	1.97 ± 0.23	200.55 ± 19.3	99.36 ± 3.86	10.98 ± 1.69
Humeri				
UC-PA (*n* = 3)	4.93 ± 1.11	111.22 ± 16.77	156.19 ± 19.39	5.02 ± 0.75
UC-PR (*n* = 3)	5.64 ± 1.44	124.12 ± 2.21	162.76 ± 14.61	5.43 ± 1.00
WB-PA (*n* = 3)	4.25 ± 0.43	114.15 ± 10.17	155.95 ± 10.60	5.88 ± 0.61
WB-PR (n = 3)	4.67 ± 0.73	105.70 ± 15.37	168.31 ± 25.07	5.01 ± 0.65
SB-PA (n = 3)	5.17 ± 2.81	108.61 ± 15.78	164.81 ± 39.55	6.07 ± 1.96
SB-PR (n = 3)	4.05 ± 0.11	110.70 ± 25.31	162.45 ± 43.98	4.87 ± 1.21

**Fig. 5:**
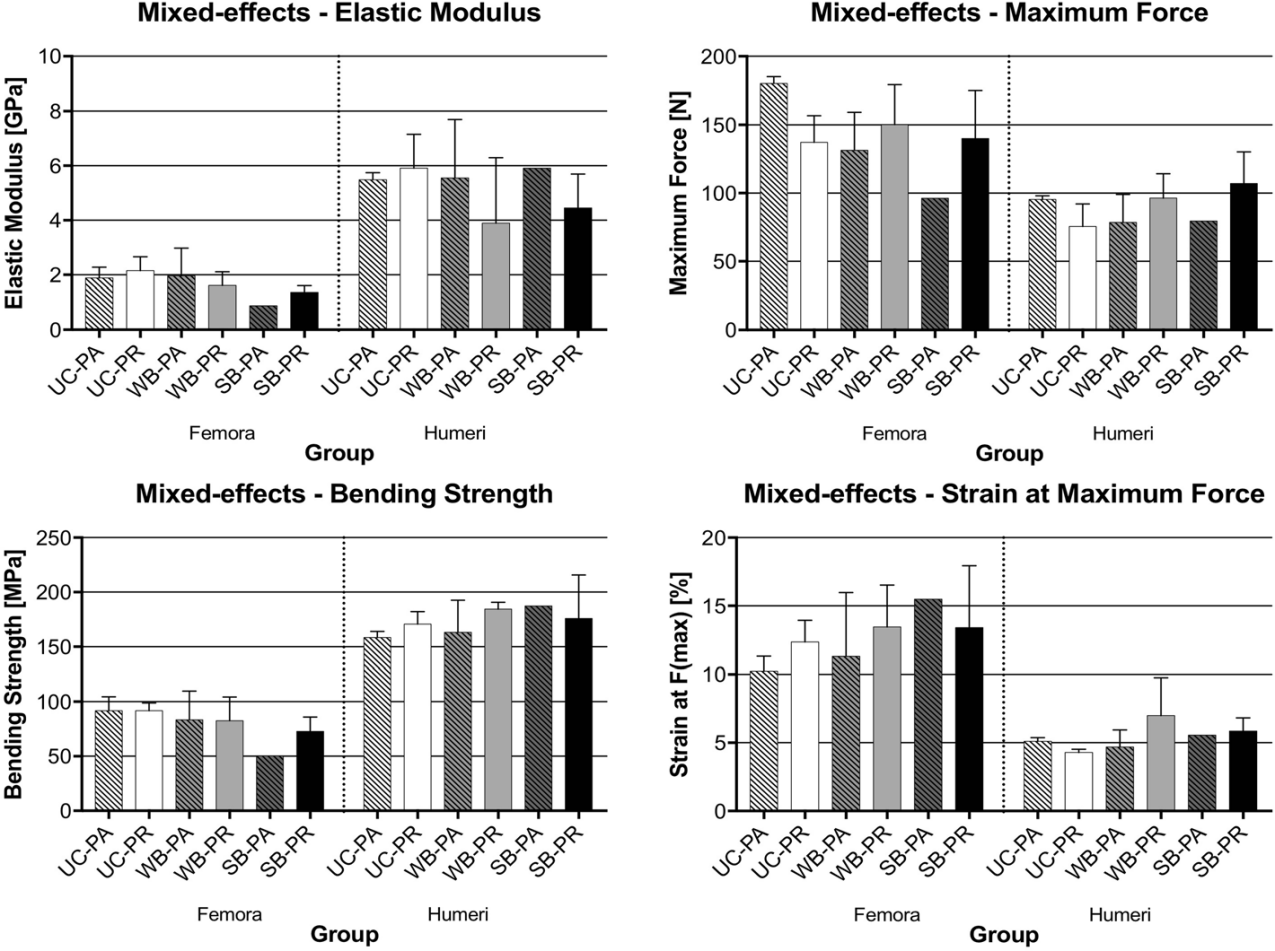
Graphs showing mean values with standard deviations for the mixed-effects group of rat femora and humeri undergoing three-point bending tests (two freezing cycles)

In the mixed-effects rats, there were also some statistically non-significant changes found between the six subgroups. In contrast to the highly-uniform group, the elastic modulus and bending strength tended to be higher in uncoated femora and humeri than coated. The SB-PA femora had lower elastic modulus, maximum force and bending strength when compared with SB-PR, which was the opposite in the highly-uniform group. Also, the elastic modulus, bending strength and strain at maximum force were higher in the UC-PR femora subgroup than in the UC-PA femora subgroup. The means for the mixed effect group are summarised in Table 3 and Fig. 5.

**Table 3:** Summary of means for mixed-effects group of rat femora and humeri undergoing three-point bending tests (two freezing cycles)

Mixed-effects Group	Elastic Modulus [GPa]	Maximum Force [N]	Bending Strength/Maximum Stress [MPa]	Strain at Maximum Force [%]
	Mean ± Standard Deviation			
Femora				
UC-PA (n = 2)	1.89 ± 0.39	180.20 ± 4.94	91.69 ± 12.81	10.24 ± 1.09
UC-PR (*n* = 2)	2.16 ± 0.51	137.2 ± 19.56	91.82 ± 7.04	12.35 ± 1.60
WB-PA (n = 3)	1.96 ± 1.02	131.40 ± 27.89	83.52 ± 26.11	11.30 ± 4.67
WB-PR (n = 3)	1.61 ± 0.50	150.20 ± 29.09	82.56 ± 21.74	13.46 ± 3.07
SB-PA (*n* = 1)	0.88	96.33	50.01	15.50
SB-PR (n = 3)	1.37 ± 0.23	140.20 ± 34.77	72.77 ± 13.17	13.4 ± 4.53
Humeri				
UC-PA (n = 2)	5.49 ± 0.26	95.39 ± 2.73	158.50 ± 5.65	5.10 ± 0.28
UC-PR (n = 2)	5.91 ± 1.25	75.64 ± 16.42	170.80 ± 11.40	4.29 ± 0.23
WB-PA (n = 3)	5.56 ± 2.13	78.66 ± 20.35	163.30 ± 29.35	4.69 ± 1.26
WB-PR (n = 3)	3.89 ± 2.41	96.42 ± 17.80	184.40 ± 6.37	6.99 ± 2.76
SB-PA (n = 1)	5.91	79.63	187.50	5.57
SB-PR (n = 3)	4.46 ± 1.24	107.00 ± 23.19	175.90 ± 39.75	5.87 ± 0.93

### SEM has shown more pronounced mechanical alterations related to slow freezing

**Fig. 6:**
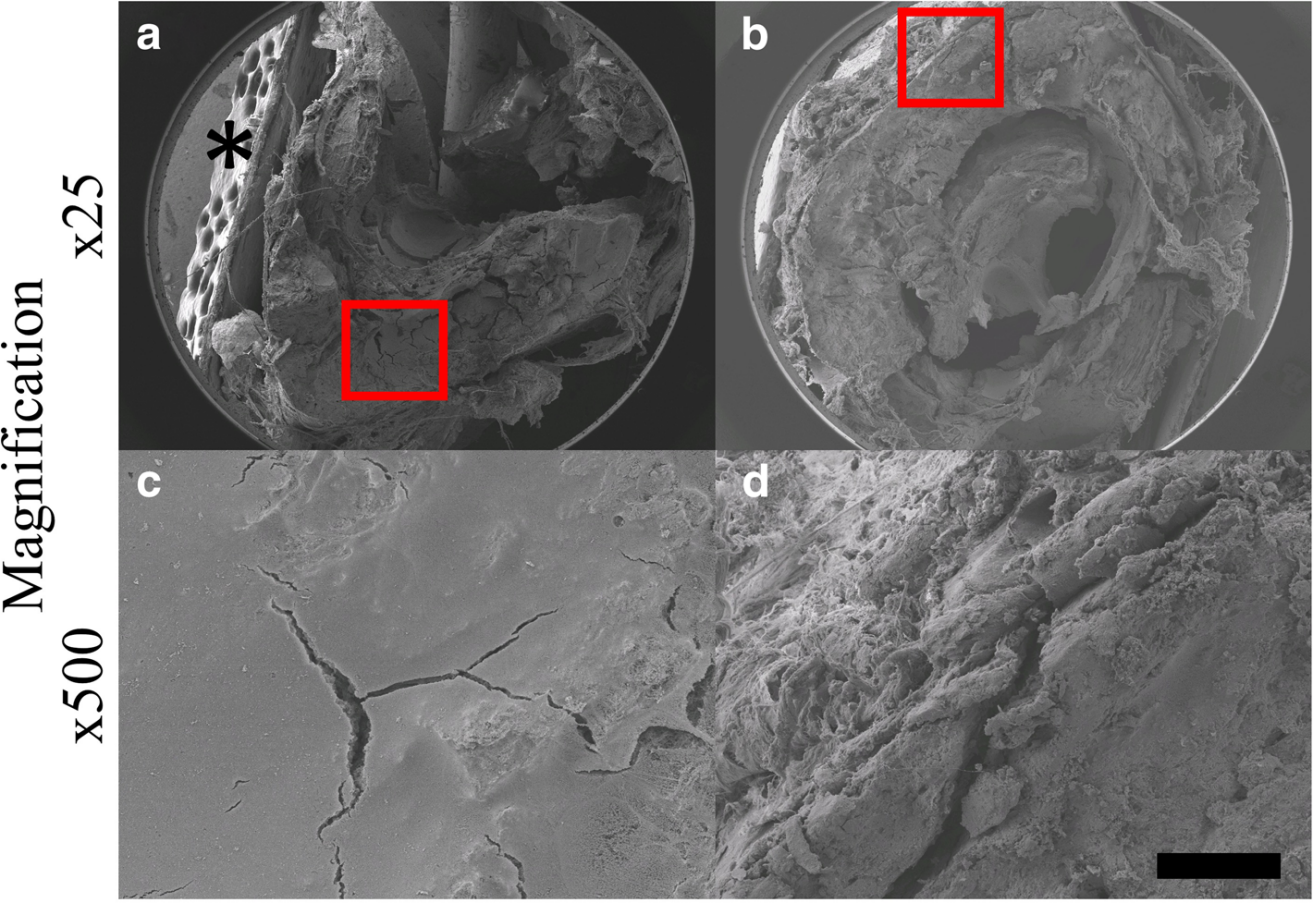
On the left (**a**, **c**), a femur from the mixed-effect group is shown with partial disruption in cortical bone integrity, indicated by crack-like cavities. On the right (**b**, **d**), a femur is depicted from the highly-uniform group showing fewer cracks, and on the edge of the bone. The asterisk (*) is to indicate the tape used to fix the bones. The bar on the bottom right corner is 1 mm for (**a**, **b**), 68 μm (**c**, **d**)

SEM revealed that there was a qualitative difference in the number and location of cracks found between the mixed-effects and highly-uniform bone groups. As seen in Fig. 6, the femur in the mixed-effects group appeared to have more mechanical damage, which was also located more internally whereas the femur from the highly-uniform group appeared to have fewer cracks, which were located peripherally. It is unclear if these cracks pre-existed prior to the mechanical tests or if they were a consequence of altered behaviour of the pre-weakened tissues.

## Discussion

This study has shown that surface coating of rat bones with commercially-available sprays for the purpose of enhancing image correlation for DIC does not significantly alter mechanical properties when tissues are tested within an adequate timeframe after coating. However, some trends were observed in the data collected; in the highly-uniform rats, the elastic modulus, maximum force, bending strength and maximum strain values were higher for the SB-PA subgroup than for UC-PA. One exception was the maximum force measured in humeri. In the mixed-effects rats, the uncoated femora tended to have slightly higher but non-significantly different elastic modulus, maximum force and bending strength.

In this study, rat bones were used as they are a reliable and highly-standardized alternative to human bones [18]. Rat bones are much thinner in diameter and therefore easier to access by chemicals until the tissues are thoroughly diffused. This results in these tissues being affected earlier than samples from larger animal models or human cadavers, thereby allowing chemically or osmotically-induced changes to be observed much quicker.

Efforts were made to have highly standardized samples, allowing inter-individual variation to be outweighed by the differences induced by fixation. This was done by having all the rats as Wistar or Sprague Dawley breeds with the same age and weight. If human samples were used, this would have led to a much greater inter-individual variation due to factors such as gender, age, and size [14]. Weight was used as a measure of maturation, alongside with age. Only male rats were used in the highly-uniform group to achieve the greatest homogeneity, but both male and female rats were used in the mixed-effects group to get close to ‘real-life’ conditions, with more statistical spread.

It was hypothesized that the solvent-based spray may increase the elastic modulus and strength in bones with periosteum still attached due to chemical or osmotic alterations, which have been discussed above. This effect was confirmed in the highly-uniform femora and humeri that had the periosteum still attached. However, the minor changes in mechanical properties were still statistically non-significant. This phenomenon was not noticed in the mixed-effects rats, but given the small sample size further quantification of this comparison was rendered not useful.

In the mixed-effects rats, the bone group that had undergone the freeze-thaw cycles, trends observed were that the uncoated femora had slightly higher values compared to the coated although still statistically non-significant. The hypothesis that this group may have shown a more pronounced difference between uncoated and coated sample values compared to the highly-uniform group was derived from the concept of ice needle formation, which has been discussed previously. However, this effect was not noticed in the mechanical experiments quantitatively, but the SEM images did show an increased number of cracks in the bone, though a causal relation at this stage remains unclear. If more cycles of freezing and thawing had occurred, the results could have been different as deterioration of mechanical properties seems to occur after five cycles of freezing and thawing [19, 20].

This is a study that examined the effects of spray coating on rat bones for the purpose of biomechanical testing with image-based deformation detection. A similar study was conducted on porcine collateral ligaments; it concluded that using methylene blue and white paint for speckling did not significantly alter the elastic responses of the soft tissues [21]. Another study investigating mouse carotid arteries with DIC also showed no significant effect on the mechanical properties when using India ink for the speckling pattern [22]. Other studies that have been conducted on bone fixation for preservation purposes have shown different results. A study examining the influence of ethanol and formaldehyde fixation on the mechanical properties of human coxal bones showed irreversible changes to the organic matrix [8]. A different study found a significant influence of chemical fixation with ethanol and formaldehyde on the mechanical properties collagen rich soft tissues such as the iliotibial tract [23]. Though these changes have been found before by these groups, such changes were not evident as a result of surface speckling, which may be a result of different samples being used or that this fixation method involved complete submersion and was long-term. In contrast, another study by the same group investigating the effect of short-term fixation with formalin or ethanol on the mechanics of human cortical bone yielded no vast alterations [11]. This is in accordance with the current study as it examined the effect of short-term fixation with commercial sprays.

### Limitations

Firstly, the sample size was relatively small, especially in the mixed-effects group; increasing the sample size may have provided more reliable results in all groups. Increasing the sample size would also have allowed for a control group for the mixed-effects that did not undergo any pre-treatment. Also, the distribution of the samples in the six subgroups could have been done differently so there would be at least two samples in each group. Moreover, this study can be extended by using more types of biological tissues to see alterations across the spectrum of biological tissues that may be used in labs. Secondly, the post-mortem delay must be considered as the samples were frozen for at least two months before they were tested. Controversial evidence suggests that freezing may generally irreversibly alter mechanical properties of unfixed bone. One group found in porcine trabecular bone that after five years, mechanical alteration occurs, whereas no significant difference exists after one year [9]. However, it is unclear if this may also apply to rat and human bone. Also, it may have been beneficial to include samples that were also female in the highly-uniform group to see if sex would affect the results. Third, the calculations for bending stress, bending strain and elastic modulus were carried out with the assumption that the bone is one solid beam with an ideal circular cross-section, and not as a hollow tube. Furthermore, mechanical alterations may possibly also have resulted from the sprays forming a film over the biological tissues. Some aerosol water-based paints contain film-forming polymers, such as acrylate polymers, which have carrier solvents such as isopropanol [24]. These paints may form a ‘shell’ around the tissues, which thereby mechanically influences the measurements by contributing to the load-bearing structures. However, the ingredients of the sprays used in this study did not contain such film-forming polymers. Even so, it was concluded from SEM that the layers seemed less influential given their minute thickness; it was difficult to even detect the attached layer of the speckling, validating this. Lastly, the exposure time for all experiments in this study was kept constant (fifteen minutes) and the influence of shorter or higher time frames remains unknown. However, there would be a high risk of measuring not only mechanical alterations by the ingredients of the sprays given the nature of ongoing dehydration processes when these tissues are subjected to environmental conditions.

## Conclusions

In conclusion, there was found to be no statistically significant difference between coated and uncoated rat femora or humeri – this holds true also for any samples that have undergone an additional freeze-thaw cycle. For the purposes of biomechanical testing using the DIC system, the spray method is recommended as it is an effective and quick method for producing a random speckle pattern [3]. Further research may be done on examining the effects of multiple freeze-thaw cycles with controls when using sprays, and on other kinds of tissues.

## Abbreviations

DIC: Digital image correlation
PA: Periosteum attached
PR: Periosteum removed
SB: Solvent based (spray)
SEM: Scanning electron microscopy
UC: Uncoated
WB: Water based (spray)
